# PKC*ζ* phosphorylates TRAF2 to protect against intestinal ischemia–reperfusion–induced injury

**DOI:** 10.1038/cddis.2017.310

**Published:** 2017-07-20

**Authors:** Wei Zhou, Jihong Yao, Guangzhi Wang, Zhao Chen, Zhenlu Li, Dongcheng Feng, Yang Li, Wasim Qasim, Wenzhi Tan, Shili Ning, Xiaofeng Tian

**Affiliations:** 1 Department of General Surgery, The Second Affiliated Hospital of Dalian Medical University, Dalian 116023, China; 2 Department of Pharmacology, Dalian Medical University, Dalian 116044, China

## Abstract

Intestinal ischemia–reperfusion (I/R) is a common clinical problem that occurs during various clinical pathological processes. Excessive apoptosis has an indispensable role in intestinal I/R injury. Tumor necrosis factor receptor-associated factor 2 (TRAF2) and PKC*ζ* have an essential role in apoptosis. Here, we aimed to investigate the effects of PKC*ζ* and TRAF2 and to explore the correlation between PKC*ζ* and TRAF2 in intestinal I/R injury. Mice were subjected to intestinal I/R injury *in vivo*. *In vitro* experiments were conducted by treating Caco-2 cells with hypoxia/reoxygenation (H/R) stimulation to simulate intestinal I/R. Intestinal tissue samples and Caco-2 cells were examined using various approaches. Intestinal I/R induced the membrane translocation and phosphorylation of PKC*ζ*. Pretreatment with the PKC*ζ* activator phosphatidylcholine remarkably attenuated gut injury by suppressing apoptosis. H/R induced PKC*ζ* to combine with TRAF2, which was phosphorylated by PKC*ζ* at Ser^55^, but not at Ser^11^, under intestinal I/R or H/R conditions. In addition, TRAF2 Ser^55^ phosphorylation increased cell survival by inhibiting cell apoptosis in the H/R model. Mechanistically, TRAF2 Ser^55^ phosphorylation promoted NF-*κ*B activation but suppressed c-Jun activation in Caco-2 cells under H/R conditions. The results of this study demonstrate that the PKC*ζ*/TRAF2 pathway represents a novel protective mechanism against intestinal I/R injury. Therefore, the PKC*ζ*/TRAF2 pathway is a novel target for potential treatments of intestinal I/R injury-related diseases.

Intestinal ischemia–reperfusion (I/R) occurs in a wide variety of clinical settings, including hemorrhagic shock, acute mesenteric ischemia, and organ transplantation,^1^ resulting in a high mortality rate.^2^ Although various therapeutic methods exist,^1^ including ischemic preconditioning, antioxidant therapy, anticomplement therapy, and antileukocyte therapy, no definite therapeutic strategy is available for intestinal I/R injury. We and others have shown that cell apoptosis is a significant contributor to intestinal I/R injury.^3, 4, 5^ However, the underlying mechanisms are not completely understood.

The protein kinase C (PKC) family consists of 12 structurally related members that function as serine/threonine kinases.^6^ PKC has essential roles in various fundamental cellular processes, including survival, proliferation, differentiation, and apoptosis.^7^ PKC*ζ* is one member of the atypical protein kinase C (aPKC) subfamily,^8^ which is expressed in intestinal stem cells and colon cancer cells.^9, 10, 11^ PKC*ζ* knockdown or dominant-negative PKC*ζ* expression increases cell apoptosis,^10, 11, 12^ suggesting that PKC*ζ* is an anti-apoptotic protein. Although one study found that PKC*ζ* was involved in MAPK activation in H9c2 cells (an embryonic rat heart-derived cell line) subjected to reoxygenation after ischemic hypoxia,^13^ the role of PKC*ζ* in intestinal I/R injury is unknown.

Tumor necrosis factor (TNF) receptor-associated factor (TRAF) proteins are adaptor molecules that associate the TNF receptor family with a variety of signaling pathways related to cell survival and cellular responses to stress.^14^ Previous studies revealed that TRAF2, which is a representative member of the TRAF family, promoted cell survival in response to TNF-*α* stimulation.^15^ In addition, TRAF2 depletion augmented hepatocyte apoptosis via Fas/CD95,^16^ implying that TRAF2 has a role in suppressing apoptosis in the liver. Research into myocardial I/R injury revealed that cardiac-restricted expression of dominant-negative TRAF2 significantly increased injury,^17^ indicating a role for TRAF2 as a cytoprotective factor in myocardial I/R. Furthermore, TRAF2 KO mice exhibited increased proinflammatory cytokine expression and colonic epithelial cell apoptosis, leading to the spontaneous development of inflammatory bowel disease (IBD).^18^ However, research has indicated that TRAF2 KO mouse embryonic fibroblasts (MEFs) are resistant to cell death induced by reactive oxygen species (ROS),^19^ suggesting that TRAF2 can promote ROS-induced cell death. Thus, TRAF2 has distinct roles in different cell systems and settings. Therefore, we speculated that TRAF2 may also be involved in intestinal I/R injury and were interested in exploring the specific effect of TRAF2 on intestinal I/R injury.

PKC*ζ* phosphorylates TRAF2 at Ser^55^ in response to TNF-*α* stimulation, which allows the phosphorylated TRAF2 to suppress apoptosis, leading to inhibition of TNF-*α*-induced cell death in MEFs.^20^ Consequently, in this study, we sought to investigate the role of the PKC*ζ*/TRAF2 signaling pathway in intestinal I/R injury.

## Results

### Intestinal I/R induces the membrane translocation and phosphorylation of PKC*ζ*

**Figure 1:**
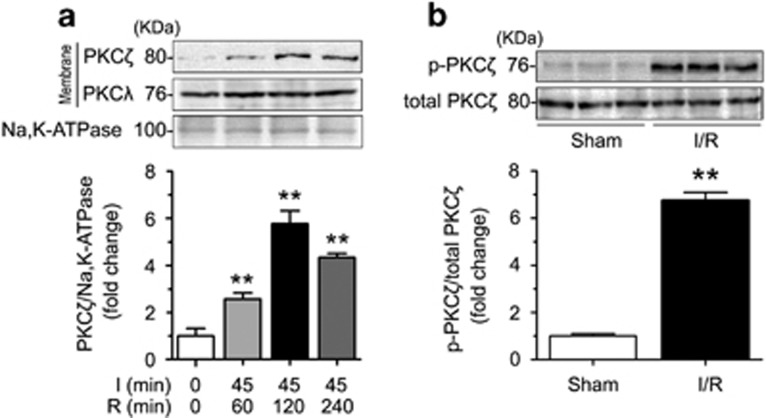
Intestinal I/R induced the membrane translocation and phosphorylation of PKC*ζ*. Mice were subjected to 45 min of ischemia followed by 60, 120, or 240 min of reperfusion. (**a**) Western blotting demonstrated PKC*ζ* and PKC*λ* expression in the membranous fractions using Na, K-ATPase as a loading control. (**b**) Western blotting demonstrated p-PKC*ζ* (Thr^410^) and total PKC*ζ* expression in the sham and 120-min reperfusion intestines. All results are expressed as the means±S.D., *n*=3 per group, **P<0.01 *versus* sham

We had previously confirmed the intestinal I/R-mediated membrane translocation and phosphorylation of PKC*β*_2_, but not PKC*β*_1_, PKCδ, or PKCε.^21^ To explore the protein expression of aPKC subfamily members (PKC*ζ* and PKC*λ*) in intestinal I/R injury, we evaluated related membrane proteins in intestinal tissues subjected to 45 min of ischemia, followed by 60, 120 or 240 min of reperfusion. The selective membrane translocation of PKC*ζ*, but not of PKC*λ* (the other member of the aPKC subfamily), was tested in the membrane fraction after different reperfusion times (Figure 1a). The results indicated that PKC*ζ*, but not PKC*β*_2_, was particularly activated by intestinal I/R.

We examined PKC*ζ* phosphorylation after a 120-min reperfusion and found a significant increase at the Thr^410^ residue, which resulted in a marked increase in the phosphorylated PKC*ζ*/total PKC*ζ* ratio (Figure 1b). Overall, these findings suggested that PKC*ζ* translocates to the plasma membrane and is phosphorylated in response to intestinal I/R.

### PKC*ζ* attenuates the gut injury induced by intestinal I/R

**Figure 2:**
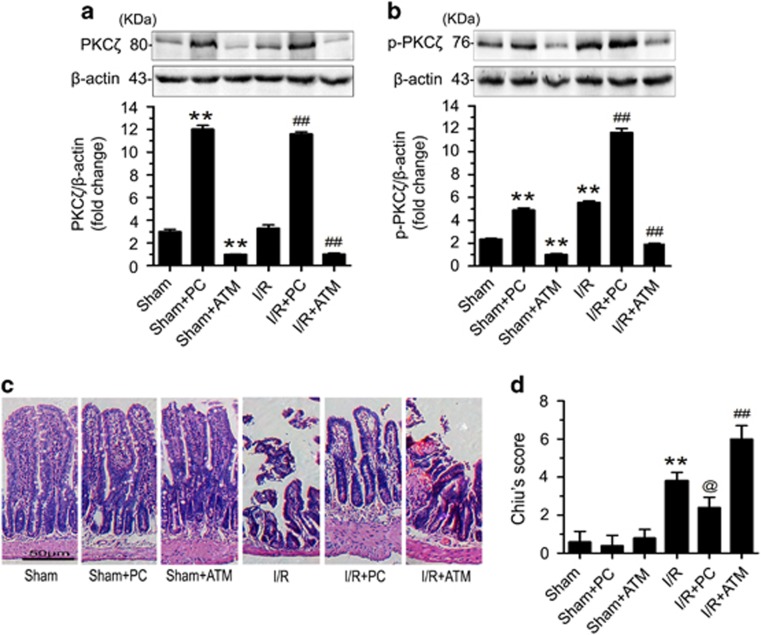
Increased PKC*ζ* activation attenuated the gut injury induced by intestinal I/R. Prior to the sham or 45-min ischemia followed by 120-min reperfusion treatment, the mice were pretreated with either normal saline, PC, or ATM. (**a**) PKC*ζ* protein expression. (**b**) p-PKC*ζ* (Thr^410^) protein expression. (**c**) Intestinal tissues were harvested and stained with hematoxylin and eosin and then examined with light microscopy at × 200 magnification. Representative images from the sham, sham+PC pretreatment, sham+ATM pretreatment, I/R, I/R+PC pretreatment, and I/R+ATM pretreatment groups. (**d**) Histological injury scores of intestines from the different groups. The data are shown as the means±S.D., *n*=8 per group, **P<0.01 *versus* sham; ^##^*P*<0.01 *versus* I/R; ^@^*P*<0.05 *versus* I/R

To assess the function of PKC*ζ* in the intestinal I/R model, we used the PKC*ζ* activator phosphatidylcholine (PC) and the PKC*ζ* inhibitor aurothiomalate (ATM).^22, 23^ First, we examined the effect of exposure of PKC*ζ* to PC or ATM. The data suggested that PC increased the expression and phosphorylation of PKC*ζ*, whereas ATM had the opposite effect (Figures 2a and b). Next, we tested the effects of PC and ATM on histological changes in intestinal tissue in response to intestinal I/R injury. Compared with the sham group, the intestinal I/R group showed severe hyperemia and a loss of intestinal villi (Figure 2c). PC clearly reduced the intestinal injury and histopathology score, however, the group treated with ATM under intestinal I/R conditions displayed visibly worsened injury and an increased histopathology score (Figures 2c and d). Collectively, these findings suggested that an increase in PKC*ζ* activation attenuated the gut injury induced by intestinal I/R.

### PKC*ζ* suppresses intestinal I/R injury by inhibiting gut apoptosis

**Figure 3:**
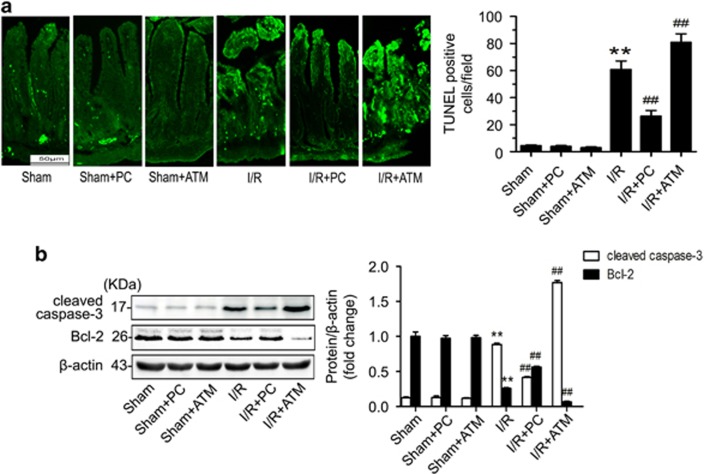
Increased activation of PKC*ζ* by PC inhibited intestinal apoptosis after intestinal I/R. (**a**) Paraffin-embedded intestinal tissue sections were stained using TUNEL and examined under light microscopy at × 200 magnification (left panels). Quantification of TUNEL staining (right panel). (**b**) cleaved caspase-3 and Bcl-2 protein expression. The data are presented as the means±S.D., *n*=3 per group, **P<0.01 *versus* sham; ^##^*P*<0.01 *versus* I/R

To define the mechanism underlying the protective effect of PKC*ζ* in intestinal I/R injury, we conducted a terminal deoxynucleotidyl transferase-mediated deoxyuridine triphosphate nick-end labeling (TUNEL) assay to evaluate if PKC*ζ* affects apoptosis. Compared with the sham group, the intestinal I/R group presented a significant increase in apoptotic cells. Conversely, pretreatment with PC resulted in a distinct reduction in the number of apoptotic cells (Figure 3a). However, the number of apoptotic intestinal cells was increased in the group treated with ATM under intestinal I/R conditions (Figure 3a). Moreover, PC apparently suppressed the expression of the pro-apoptotic protein cleaved caspase-3 and increased the expression of the anti-apoptotic protein Bcl-2 (Figure 3b). Overall, the data suggested that an increase in PKC*ζ* activation could alleviate the apoptosis induced by intestinal I/R.

### PKC*ζ* colocalizes with TRAF2 after H/R and TRAF2 is phosphorylated in response to intestinal I/R

**Figure 4:**
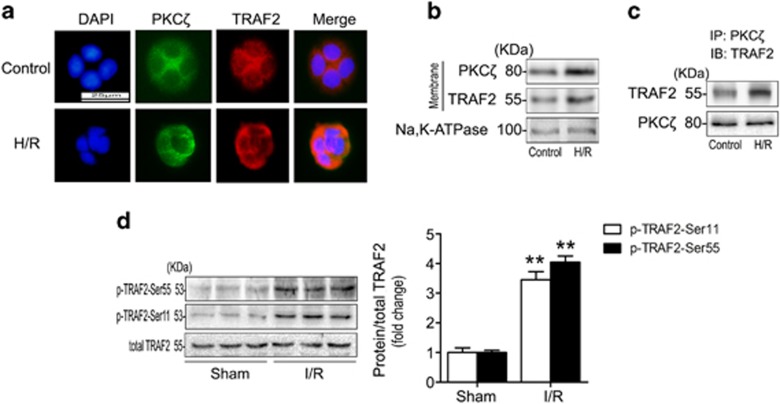
H/R induced PKC*ζ* to combine with TRAF2, and TRAF2 was phosphorylated in response to intestinal I/R. (**a**) Caco-2 cells were subjected to H/R, fixed with 4% paraformaldehyde, and stained with a rabbit polyclonal TRAF2 (red, 1:100 dilution) antibody in combination with a mouse polyclonal PKC*ζ* antibody (green, 1:50 dilution); 4,6-diamino-2-phenylindole (blue, 1 g/ml) was used to visualize the nuclei. The cells were examined using light microscopy at × 400 magnification. (**b**) Western blotting demonstrated the expression of PKC*ζ* and TRAF2 in the membranous fractions using Na, K-ATPase as a loading control. (**c**) Membrane fractions were isolated from Caco-2 cells. Equal amounts of PKC*ζ* were immunoprecipitated (IP) with a mouse polyclonal PKC*ζ* antibody and immunoblotted (IB) with a rabbit polyclonal antibody targeting TRAF2. (**d**) p-TRAF2-Ser55 and p-TRAF2-Ser11 protein expression was evaluated using total TRAF2 as a loading control. All results are expressed as the means±S.D., *n*=3 per group, **P<0.01 *versus* sham

To explore the mechanism by which PKC*ζ* phosphorylation alleviated the apoptosis induced by intestinal I/R, human Caco-2 cells were treated with hypoxia/reoxygenation (H/R) to mimic the intestinal I/R model. We found that PKC*ζ* and TRAF2 were recruited to the plasma membrane and combined with each other after H/R treatment (Figure 4a). Western blotting further verified the membrane translocation of PKC*ζ* and TRAF2 in Caco-2 cells under H/R conditions (Figure 4b). Next, we conducted a co-immunoprecipitation experiment to examine the association between PKC*ζ* and TRAF2. Membranes isolated from Caco-2 cells in the absence of H/R (control) showed colocalization of PKC*ζ* and TRAF2 in the same immunocomplex, whereas H/R increased the appearance of TRAF2 in the PKC*ζ* immunoprecipitates (Figure 4c).

We also examined TRAF2 phosphorylation in response to intestinal I/R. The results suggested that TRAF2 was phosphorylated at both Ser^55^ and Ser^11^, but the total TRAF2 expression level did not change (Figure 4d). Therefore, our data showed that TRAF2 was recruited to the plasma membrane and combined with PKC*ζ* in Caco-2 cells under H/R conditions, and TRAF2 was activated through phosphorylation at both Ser^55^ and Ser^11^ in intestinal I/R.

### TRAF2 is phosphorylated by PKC*ζ* at Ser^55^ but not at Ser^11^ in response to intestinal I/R or H/R

**Figure 5:**
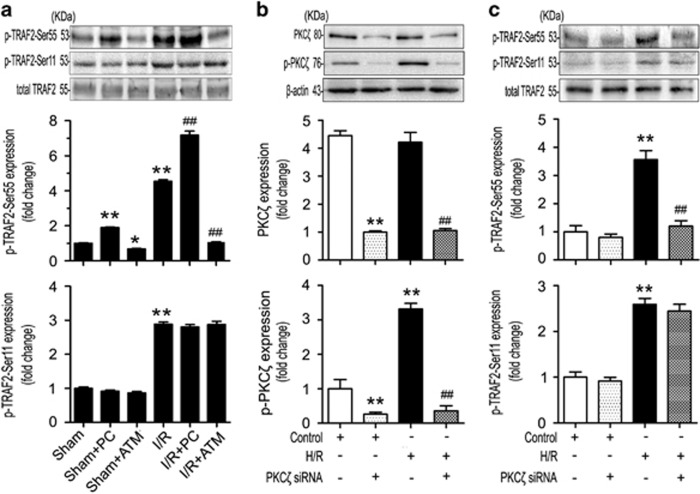
TRAF2 was phosphorylated by PKC*ζ* at Ser^55^ but not at Ser^11^ under intestinal I/R or H/R conditions. (**a**) p-TRAF2-Ser55 and p-TRAF2-Ser11 expression using total TRAF2 as a loading control in the intestinal tissues of mice subjected to sham, sham+PC pretreatment, sham+ATM pretreatment, I/R, I/R+PC pretreatment, and I/R+ATM pretreatment. (**b**) PKC*ζ* and p-PKC*ζ* and (**c**) p-TRAF2-Ser55 and p-TRAF2-Ser11 expression in Caco-2 cells treated with PKC*ζ* siRNA under untreated or H/R conditions. Values are represented as the means±S.D., *n*=3 per group, **P<0.01 *versus* sham; *P<0.05 *versus* sham; ^##^*P*<0.01 *versus* I/R

To determine whether both Ser^55^ and Ser^11^ of TRAF2 were phosphorylated by PKC*ζ*, we performed *in vivo* and *in vitro* experiments. The results of the *in vivo* experiment showed that p-TRAF2-Ser55 expression was markedly increased by pretreatment with PC, whereas TRAF2 phosphorylation at Ser^55^ was reduced in the groups treated with ATM (Figure 5a). However, no effect on p-TRAF2-Ser11 expression was observed regardless of pretreatment with PC or ATM (Figure 5a).

To confirm that PKC*ζ* was involved in TRAF2 activation at Ser^55^ but not Ser^11^, we knocked down endogenous PKC*ζ* in Caco-2 cells using a typical siRNA approach (Figures 5b and c). The siRNA-mediated knockdown of PKC*ζ* significantly suppressed the expression of PKC*ζ* and p-PKC*ζ* in Caco-2 cells (Figure 5b). Notably, knockdown of PKC*ζ* also reduced TRAF2 phosphorylation at Ser^55^ but had no effect on phosphorylation at Ser^11^ in Caco-2 cells under H/R conditions (Figure 5c). Consequently, TRAF2 was targeted at Ser^55^, but not at Ser^11^, by PKC*ζ* after intestinal I/R or H/R.

### TRAF2 Ser^55^ phosphorylation attenuates cell death by inhibiting cell apoptosis

**Figure 6:**
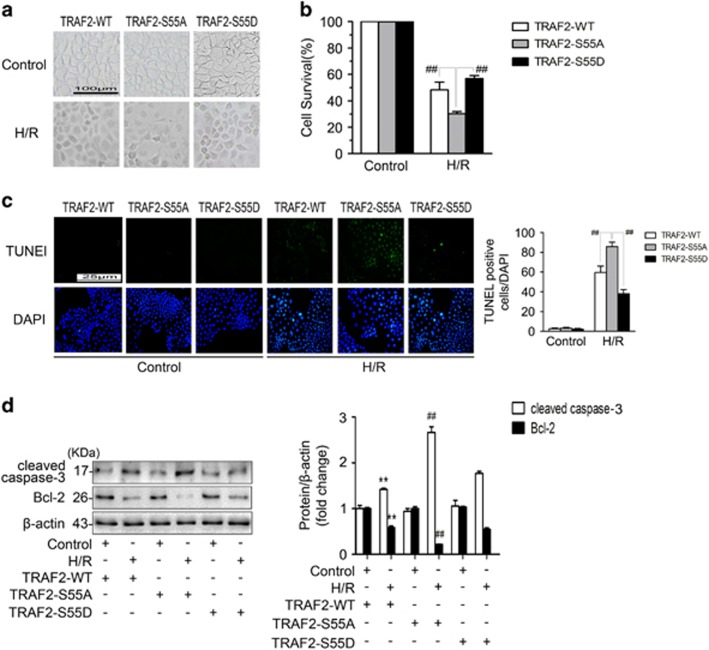
TRAF2 Ser^55^ phosphorylation increased cell survival by inhibiting cell apoptosis in response to H/R. Caco-2 cells were transfected with TRAF2-WT, TRAF2-S55A, and TRAF2-S55D and then were subjected to no treatment or H/R. (**a**) The cellular morphology and structure of Caco-2 cells by bright image (× 100 magnification) investigation. (**b**) CCK-8 was used to examine cell survival. (**c**) Caco-2 cells were treated as described above, stained using TUNEL and examined under light microscopy at × 400 magnification (left panels). Relative apoptotic rates are represented as TUNEL-positive cells/DAPI (right panel). (**d**) Western blotting demonstrated the expression of cleaved caspase-3 and Bcl-2. *β*-actin was included as a loading control. The data are shown as the means±S.D., *n*=6 per group, **P<0.01 *versus* control+TRAF2-WT; ^##^*P*<0.01 *versus* H/R+TRAF2-WT and H/R+TRAF2-S55D

To elucidate the mechanism by which TRAF2 Ser^55^ phosphorylation attenuated cell death in Caco-2 cells under H/R conditions, we constructed two phospho-mutant TRAF2 plasmids: TRAF2-S55A, in which Ser55 was mutated to alanine to abolish phosphorylation, and TRAF2-S55D, in which Ser55 was mutated to aspartic acid to mimic phosphorylation. By performing western blotting, we found that the expression of p-TRAF2-Ser55 was markedly lower in Caco-2 cells transfected with TRAF2-S55A than that of the empty plasmid, while TRAF2-S55D transfection played the opposite role (Supplementary Figure S1). Caco-2 cells were transfected with TRAF2-WT, TRAF2-S55A, or TRAF2-S55D and then subjected to H/R or left untreated. Based on the cell morphology and cell viability assay, pretreatment with TRAF2-S55D reduced cell death (Figures 6a and b). To clarify the underlying mechanism, we conducted a TUNEL assay. The results showed that transfection with TRAF2-S55D reduced cell apoptosis compared with the other groups (Figure 6c). Moreover, the western blotting results confirmed that Caco-2 cells treated with TRAF2-S55D exhibited reduced cleaved caspase-3 expression and increased Bcl-2 expression (Figure 6d). In summary, the data suggested that TRAF2 Ser^55^ phosphorylation increased cell survival by inhibiting cell apoptosis in Caco-2 cells under H/R conditions.

### TRAF2 Ser^55^ phosphorylation promotes NF-*κ*B activation but suppresses c-Jun activation under H/R conditions

**Figure 7:**
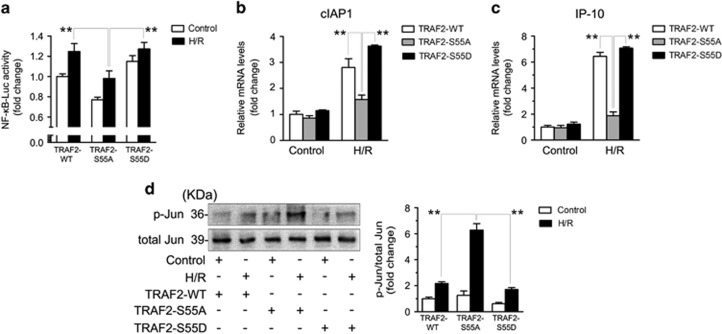
TRAF2 Ser^55^ phosphorylation increased the activation of NF-*κ*B but decreased the activation of c-Jun in response to H/R. Caco-2 cells were transfected with TRAF2-WT, TRAF2-S55A, and TRAF2-S55D and then subjected to H/R or left untreated. (**a**) NF-*κ*B activity was measured via luciferase gene reporter assays. (**b**) and (**c**) RT-PCR analysis of cIAP1 and IP-10 expression. (**d**) Western blotting demonstrated p-Jun (Thr^91^+Thr^93^) and total Jun expression. The data are shown as the means±S.D., *n*=6 per group, **P<0.01 *versus* the H/R+TRAF2-WT and H/R+TRAF2-S55D

To assess the underlying anti-apoptotic mechanism of TRAF2 Ser^55^ phosphorylation in Caco-2 cells under H/R conditions, Caco-2 cells were transfected with TRAF2-WT, TRAF2-S55A, or TRAF2-S55D and then subjected to H/R or left untreated. Luciferase reporter gene assays in Caco-2 cells revealed that TRAF2-S55D transfection increased NF-*κ*B activity compared with TRAF2-S55A transfection (Figure 7a). To further evaluate the effect of TRAF2 Ser^55^ phosphorylation on NF-*κ*B activation, we examined the expression of NF-*κ*B-related target genes by real-time PCR in Caco-2 cells under untreated or H/R conditions. Consistent with the luciferase reporter gene assay result, Caco-2 cells transfected with TRAF2-S55D had obviously promoted expression of cIAP1 and IP-10 in response to H/R compared with cells transfected with TRAF2-S55A (Figures 7b and c). In contrast, western blotting showed that TRAF2-S55D transfection reduced the phosphorylation of c-Jun compared with TRAF2-S55A transfection (Figure 7d). Taken together, these data suggested that phosphorylation of TRAF2 at Ser^55^ promoted NF-*κ*B activation but suppressed c-Jun activation in Caco-2 cells under H/R conditions.

## Discussion

Intestinal ischemia and subsequent reperfusion injuries give rise to oxidative stress and the release of inflammatory mediators,^24, 25^ such as TNF-*α* and IL-6,^5, 26^ resulting in a high mortality rate that ranges from 70 to 80%.^27, 28^ We previously verified that intestinal I/R induced the expression of ROS and TNF-*α*.^4, 21^ Apoptosis has an essential role in intestinal I/R, although the molecular basis is complex.^29^ The protective effects of PKC*ζ* in myocardial I/R have been confirmed in *in vivo* experiments.^13^ In addition, the protective role of TRAF2 in myocardial I/R has been verified in *in vitro* experiments.^17^ However, the underlying mechanisms remain unclear. The results of the present study demonstrated that the PKC*ζ*/TRAF2 signaling pathway alleviates intestinal I/R injury by suppressing apoptosis.

Although PKC*β*_1_, PKCδ, and PKCε protein expression was examined in intestinal tissues, only the level of PKC*β*_2_ expression increased in response to intestinal I/R.^21^ Similarly, in this study, we found that PKC*ζ*, but not PKC*λ*, was selectively enhanced in response to reperfusion for various times (Figure 1a). According to research on the TC10 pathway, TC10 recruits PKC*ζ*/*λ* to the plasma membrane after insulin stimulation.^30^ However, one investigation of myocardial I/R injury suggested that PI3K activated PKC*ζ* and then translocated to the nucleus from the cytoplasm.^13^ In our study, PKC*ζ* displayed membrane translocation in response to intestinal I/R (Figure 1a). These data suggested that PKC*ζ* presents distinct tissue-specific functions.

PKC*ζ* is activated mainly through three different mechanisms: phosphorylation at Thr^410^, auto-phosphorylation at Thr^560^, and release of the pseudo-substrate sequence via a conformational change.^31^ Recent research has indicated that lyso-PC, a product of the hydrolysis of PC, could effectively activate PKC*ζ* in melanocytes, NK cells, and intestinal tissue.^23, 32, 33^ Specifically, lyso-PC stimulates PKC*ζ* activation in melanocytes or NK cells through the mechanism involving phosphorylation or auto-phosphorylation, respectively.^32, 33^ In the results presented here, phosphorylation of PKC*ζ* was increased after treatment of mice with PC (Figure 2b). To further evaluate the effect of PKC*ζ* on intestinal I/R injury, we treated mice with the PKC*ζ* inhibitor ATM, which is currently in phase I clinical testing for non-small cell lung cancer (NSCLC).^34^ The PB1 functional domain has a crucial role in regulating the activity of the aPKC subfamily members.^35, 36^ in addition, the PB1 domain is intimately related to cell proliferation and cell survival.^37, 38^ ATM has an anti-tumor role in pancreatic cancer and NSCLC through the highly selective targeting of Cys-68 within the PB1 domain of PKC*ζ* or Cys-69 within the PB1 domain of PKCι.^22, 39^ Our results showed that ATM pretreatment could decrease the expression of PKC*ζ* (Figure 2a). These data indicated that PC and ATM were evidently effective in regulating the activity of PKC*ζ* in the intestine.

The TRAF family consists of six members. TRAF2 is the most extensively studied member in terms of function and structure.^14^ Moreover, TRAF2 activation and regulation are complex events. Phosphorylation of TRAF2 occurs at diverse residues, including Ser^55^, Ser^11^, and Thr^117^, which is mediated by PKC*ζ*, the IKKε kinases, and PKCδ/ε, respectively.^20, 40, 41^ In this study, we found that Ser^55^ and Ser^11^ of TRAF2 were both phosphorylated in the intestinal I/R model (Figure 4d). Interestingly, through the activation or knockdown of PKC*ζ* *in vivo* and *in vitro*, we verified that PKC*ζ* phosphorylated TRAF2 at Ser^55^, but not at Ser^11^, in response to intestinal I/R or H/R (Figure 5). A previous study indicated that TNF-*α* stimulation induced PKC*ζ* to directly phosphorylate TRAF2 at Ser^55^.^20^ In parallel, our immunofluorescence and co-immunoprecipitation assays showed that H/R stimulation of Caco-2 cells induced PKC*ζ* to combine with TRAF2 (Figures 4a and c).

TRAF2 is involved in a variety of clinical diseases, including different types of cancer,^42, 43^ IBD,^18^ and multiple pathologically induced myocardial I/R injury.^17^ However, the effect of TRAF2 on cell survival varies under diverse conditions. Some studies have suggested that TRAF2 reduces the cell death induced by TNF-*α* stimulation.^15, 20, 44^ In contrast, one study claimed that TRAF2 significantly promoted ROS-induced cell death.^19^ In this study, we showed that TRAF2 Ser^55^ phosphorylation clearly attenuated cell death (Figures 6a and b), suggesting that TRAF2 played a protective role in Caco-2 cells under H/R conditions. The TUNEL assay and evaluation of cleaved caspase-3 and Bcl-2 expression ultimately demonstrated that the mechanism underlying the protective effects of TRAF2 involved the suppression of cell apoptosis (Figures 6c and d). Therefore, we believe that TRAF2 can decrease cell death by inhibiting apoptosis in Caco-2 cells subjected to H/R.

The mechanism underlying TRAF2-mediated suppression of apoptosis is complex. Some research has confirmed that TRAF2 functions in the regulation of the NF-*κ*B and c-Jun signaling pathways.^15, 20^ Moreover, NF-*κ*B has a vital role in inhibiting apoptosis,^45^ whereas c-Jun is crucial in the pro-apoptosis cascade.^46^ The anti-apoptotic effect of NF-*κ*B may be related to Bcl-2, a key target gene of NF-*κ*B,^47, 48^ and overexpression of Bcl-2 can rescue cells from I/R- and H/R-induced apoptosis.^49, 50^ Noteworthily, a recent study about the sexual differentiation of the anteroventral periventricular nucleus (AVPV), which is thought to occur through apoptosis, confirmed that TRAF2-inhibiting protein could downregulate the TNF-*α*-TNF receptor 2-NF-*κ*B-Bcl-2 pathway to inhibit cell survival in the male AVPV.^51^ However, there is no consensus regarding the specific effects of TRAF2 on NF-*κ*B and c-Jun activation. Related research has indicated that TRAF2 could promote the activation of both NF-*κ*B and c-Jun in HEK293 cells.^52^ However, other studies asserted that TRAF2 is an activator of NF-*κ*B but inhibits c-Jun in MEFs subjected to TNF-*α* stimulation.^15, 20^ In the present study, we demonstrated that TRAF2 Ser^55^ phosphorylation could promote NF-*κ*B activation but suppress c-Jun activation in Caco-2 cells subjected to H/R (Figure 7). Therefore, our results verified that TRAF2 Ser^55^ phosphorylation mediated suppression of H/R-induced apoptosis in Caco-2 cells may be related to promoting the activation of NF-*κ*B and inhibiting the activation of c-Jun.

In conclusion, the present study showed that the PKC*ζ*/TRAF2 signaling pathway has an essential protective role in response to intestinal I/R injury. Upon intestinal I/R insult, PKC*ζ* transfers to the cell membrane and recruits and phosphorylates TRAF2 at Ser^55^, but not at Ser^11^. Subsequently, TRAF2 Ser^55^ phosphorylation activates NF-*κ*B but inhibits c-Jun to attenuate cell apoptosis, leading to protection against the injury induced by intestinal I/R. Therefore, we believe that the PKC*ζ*/TRAF2 signaling pathway represents a promising therapeutic target for intestinal I/R injury and related clinical diseases.

## Materials and Methods

### Murine model of intestinal I/R and treatment

Male C57BL/6 mice (aged 8 weeks) weighing 20±2 g were obtained from the Animal Center of Dalian Medical University (Dalian, China). The mice were provided suitable food and water and were housed in an environment with controlled humidity, temperature, and light (12 h light/dark). The intestinal I/R model was generated as previously described.^21, 24^ In brief, the mice were administered anesthesia via intraperitoneal injection of pentobarbital (40 mg/kg body weight) before midline laparotomy. Next, an atraumatic clip was used to occlude the superior mesenteric artery for 45 min, and then 90, 120, or 240 min of reperfusion was performed. Normal saline and PC (0.3 mM, Aladdin, Shanghai, China) were infused intraduodenally at 0.2 ml/h for 4 h prior to surgery.^23^ Normal saline and ATM (60 mg/kg/day, Sigma-Aldrich, St. Louis, MO, USA) were injected intraperitoneally for 7 consecutive days prior to surgery.^22^ The animal experiments were conducted in accordance with the Guidelines for the Care and Use of Laboratory Animals. All animal procedures were approved by the Institutional Ethics Committee of Dalian Medical University (Dalian, China).

### Cell culture and H/R model

Caco-2 cells were cultured in a humidified incubator maintained at 37 °C and 5% CO_2_ in Dulbecco's modified Eagle's medium supplemented with 1% non-essential amino acids, 10% fetal bovine serum, and 1% glutamide (Gibco, Carlsbad, CA, USA). To generate the *in vitro* I/R model, cells were incubated for 12 h in a microaerophilic system (Thermo, Marietta, GA, USA) containing 5% CO_2_ and 1% O_2_ balanced with 94% N_2_ gas. Then, the cells were cultured for 6 h under normoxic conditions to allow reoxygenation.

### Transient transfection of siRNA

Caco-2 cells were transfected with a PKC*ζ* siRNA (si-PKC*ζ*) or a negative control siRNA (both obtained from GenePharma, Shanghai, China) using Lipofectamine 3000 (Invitrogen, Shanghai, China). The si-PKC*ζ* had the following sequences: forward (F) 5′-GCAAACUGCUGGUCCAUAATT-3′ and reverse (R) 5′-UUAUGGACCAGCAGUUUGCTT-3′. The negative control siRNA had the following sequences: forward (F) 5'-ACGUGACACGUUCGGAGAATT-3' and reverse (R) 5'-UUCUCCGAACGUGUCACGUTT-3'. All transfection processes were performed according to the manufacturer's instructions.

### Plasmid construction and transient transfection

The TRAF2-WT plasmid, phospho-mutant TRAF2 plasmids (TRAF2-Ser55A and TRAF2-Ser55D) and the empty vector plasmid were synthesized by GenePharma. Caco-2 cells were transfected with 2 *μ*g of the TRAF2-WT, TRAF2-Ser55A or TRAF2-Ser55D plasmid or the empty vector plasmid using Lipofectamine 3000 according to the manufacturer's instructions.

### Western blotting analysis

Membranous or total proteins were extracted from intestinal tissue and Caco-2 cells using a commercial protein isolation kit (KeyGEN Biotech, Nanjing, China). Equal amounts of protein were analyzed using 10–15% SDS-PAGE (Bio-Rad, Hercules, CA, USA) and then were transferred to PVDF membranes (Millipore, Bedford, MA, USA). The membranes were incubated overnight at 4 °C with the following corresponding primary antibodies: PKC*ζ*, phospho-PKC*ζ* (Thr^410^), and TRAF2 (Santa Cruz Biotechnology, Santa Cruz, CA, USA); phospho-TRAF2-Ser11 (Cell Signaling Technologies, CST, MA, USA); PKC*λ* (R&D Systems, MN, USA); cleaved caspase-3, Bcl-2, Na, K-ATPase, and Jun (Proteintech, Wuhan, China); phospho-Jun (Thr^91^+Thr^93^) (BIOS, Beijing, China); *β*-actin (ZSGB-BIO, Beijing, China); and a phospho-TRAF2-Ser55 antibody that was custom made by GL Biochem (Shanghai, China). Then, the cells were incubated with homologous secondary antibodies for 2 h at 37 °C. The bands were exposed using enhanced chemiluminescence-plus reagents (Beyotime Institute of Biotechnology). The images were documented using a BioSpectrum-410 multispectral imaging system, and the signals were analyzed using a Gel-Pro Analyzer (Version 5.0; Media Cybernetics, Rockville, MD, USA).

### Immunofluorescence

Caco-2 cells were fixed using 4% paraformaldehyde for 30 min, washed three times with PBS, permeabilized with 0.2% Triton X-100 for 10 min, and then blocked with 2% bovine serum albumin in PBS at 37 °C for 30 min. The specimen slides were incubated with the primary anti-PKC*ζ* or anti-TRAF2 antibody at 4 °C overnight, subsequently washed three times with PBS, and then incubated with FITC- (ZSGB-BIO) and Alexa Fluor 594-conjugated secondary antibodies (Proteintech) at 37 °C for 1 h. After additional washes with PBS, the specimen slides were counterstained with the nuclear stain 4,6-diamidino-2-phenylindole (DAPI; Beyotime, Shanghai, China) at room temperature for 10 min. An 80i Nikon microscope (Tokyo, Japan) was used to examine the immunofluorescent images.

### Co-immunoprecipitation

Membranes were extracted from the Caco-2 cells as described above. An equal amount of anti-PKC*ζ* antibody was added to 500 *μ*g of protein and gently shaken at 4 °C overnight. Immunocomplexes were acquired by adding 40 *μ*l of protein A+G agarose beads (Beyotime Institute of Biotechnology, Shanghai, China); then, the mixtures were gently shaken at 4 °C for 4 h. The mixture was centrifuged at 1000 × *g* for 5 min at 4 °C, and then the supernatant was discarded. The sediment was washed five times using ice-cold PBS. To separate the immunocomplexes from the beads, the immunocomplexes were boiled in sodium dodecyl sulfate sample buffer for 5 min. Then, the specimens were examined via western blotting with anti-PKC*ζ* and anti-TRAF2 antibodies according to the manufacturer’s instructions.

### Histological and TUNEL staining

For the histological and TUNEL analyses, intestinal tissue was fixed in 4% formalin and then paraffin-embedded. Sections 4 *μ*m in thickness were stained using hematoxylin and eosin. The histopathological scores of the intestinal tissues were determined according to the method of Chiu.^53^ TUNEL staining was performed using an apoptosis detection kit (Roche, Branchburg, NJ, USA) according to the manufacturer’s instructions.

### Luciferase gene reporter assays

Caco-2 cells were cotransfected with NF-*κ*B firefly luciferase reporter plasmid (GenePharma, Shanghai, China) and TRAF2-WT, TRAF2-Ser55A, TRAF2-Ser55D or an empty vector plasmid (2 *μ*g/well) using Lipofectamine 3000. Reporter assays were performed 36 h after transfection. Luciferase activity was tested with a Double-Luciferase Reporter Assay Kit (TransGen Biotech, Beijing, China) using the Dual-Light Chemiluminescent Reporter Gene Assay System (Berthold, Germany) and was normalized to Renilla luciferase activity.

### Real-time PCR

Total RNA was extracted from Caco-2 cells using TRIzol (Takara, Dalian, China). cDNA was synthesized using the TransScript All-in-One First-Strand cDNA Synthesis SuperMix for qPCR Kit (TransGen Biotech), and target cDNA was amplified with the corresponding primers (Invitrogen). The primer sequences were as follows: cIAP1, forward (F) 5-GCCTGATGCTGGATAACTGG-3 and reverse (R) 5′-GGCGACAGAAAAGTCAATGG-3′ IP-10, forward (F) 5′-CTACTGAGGTGCTATGTTCTTAGTG-3′ and reverse (R) 5′-GTACCCTTGGAAGATGGGAAAG-3′ *β*-actin, forward (F) 5′-TCCACGAAACTACCTTCAAC-3′ and reverse (R) 5′-TTTAGGATGGCAAGGGAC-3′. The resulting cDNA was then subjected to quantitative real-time PCR using a TransStart Top Green qPCR SuperMix kit (TransGen Biotech). A 7500 Fast Real-time PCR System (Applied Biosystems) was used to analyze the specimens.

### Cell viability assay

The tetrazolium salt Cell Counting Kit-8 (Dojindo Molecular Technologies, Inc., Tokyo, Japan) was used to determine cell viability according to the manufacturer's protocol.

### Statistical analysis

Values are presented as the means±S.D. Comparisons between two groups were performed using a two-tailed Student's *t*-test. One-way analysis of variance and the Student-Newman-Keuls test were used to compare the means among multiple groups. All data analyses were performed with GraphPad Prism 5.0 (GraphPad Prism Software, La Jolla, CA, USA). *P*<0.05 was considered significant.

## References

[bib1] Collard CD, Gelman S. Pathophysiology, clinical manifestations, and prevention of ischemia-reperfusion injury. Anesthesiology 2001; 94: 1133–1138. 11465607 10.1097/00000542-200106000-00030

[bib2] Mallick IH, Yang WX, Winslet MC, Seifalian AM. Ischemia-reperfusion injury of the intestine and protective strategies against injury. Digest Dis Sci 2004; 49: 1359–1377. 15481305 10.1023/b:ddas.0000042232.98927.91

[bib3] Ikeda H, Suzuki Y, Suzuki M, Koike M, Tamura J, Tong J et al. Apoptosis is a major mode of cell death caused by ischaemia and ischaemia/reperfusion injury to the rat intestinal epithelium. Gut 1998; 42: 530–537. 9616316 10.1136/gut.42.4.530 PMC1727054

[bib4] Wang GZ, Yao JH, Li ZL, Zu G, Feng DC, Shan W et al. miR-34a-5p inhibition alleviates intestinal ischemia/reperfusion-induced reactive oxygen species accumulation and apoptosis via activation of SIRT1 signaling. Antioxid Redox Signal 2016; 24: 961–973. 26935288 10.1089/ars.2015.6492

[bib5] Zhang F, Hu Y, Xu X, Zhai X, Wang G, Ning S et al. Icariin protects against intestinal ischemia-reperfusion injury. J Surg Res 2015; 194: 127–138. 25472572 10.1016/j.jss.2014.10.004

[bib6] Xiao H, Liu M. Atypical protein kinase C in cell motility. Cell Mol Life Sci 2013; 70: 3057–3066. 23096778 10.1007/s00018-012-1192-1 PMC11113714

[bib7] Breitkreutz D, Braiman-Wiksman L, Daum N, Denning MF, Tennenbaum T. Protein kinase C family: on the crossroads of cell signaling in skin and tumor epithelium. J Cancer Res Clin 2007; 133: 793–808. 10.1007/s00432-007-0280-3 PMC12160839 17661083

[bib8] Moscat J, Diaz-Meco MT, Wooten MW. Of the atypical PKCs, Par-4 and p62: recent understandings of the biology and pathology of a PB1-dominated complex. Cell Death Differ 2009; 16: 1426–1437. 19713972 10.1038/cdd.2009.119 PMC3975918

[bib9] Llado V, Nakanishi Y, Duran A, Reina-Campos M, Shelton PM, Linares JF et al. Repression of intestinal stem cell function and tumorigenesis through direct phosphorylation of beta-catenin and Yap by PKCzeta. Cell Rep 2015; pii: S2211-1247(15)00008-X. 10.1016/j.celrep.2015.01.007 PMC4524805 25660024

[bib10] Luna-Ulloa LB, Hernandez-Maqueda JG, Santoyo-Ramos P, Castaneda-Patlan MC, Robles-Flores M. Protein kinase C zeta is a positive modulator of canonical Wnt signaling pathway in tumoral colon cell lines. Carcinogenesis 2011; 32: 1615–1624. 21859831 10.1093/carcin/bgr190

[bib11] Umemori Y, Kuribayashi K, Nirasawa S, Kondoh T, Tanaka M, Kobayashi D et al. Protein kinase C zeta regulates survivin expression and inhibits apoptosis in colon cancer. Int J Oncol 2014; 45: 1043–1050. 24920238 10.3892/ijo.2014.2489

[bib12] Ghosh PM, Bedolla R, Mikhailova M, Kreisberg JI. RhoA-dependent murine prostate cancer cell proliferation and apoptosis: role of protein kinase Czeta. Cancer Res 2002; 62: 2630–2636. 11980660

[bib13] Mizukami Y, Kobayashi S, Uberall F, Hellbert K, Kobayashi N, Yoshida K. Nuclear mitogen-activated protein kinase activation by protein kinase czeta during reoxygenation after ischemic hypoxia. J Biol Chem 2000; 275: 19921–19927. 10777509 10.1074/jbc.M907901199

[bib14] Bradley JR, Pober JS. Tumor necrosis factor receptor-associated factors (TRAFs). Oncogene 2001; 20: 6482–6491. 11607847 10.1038/sj.onc.1204788

[bib15] Zhang L, Blackwell K, Altaeva A, Shi Z, Habelhah H. TRAF2 phosphorylation promotes NF-kappaB-dependent gene expression and inhibits oxidative stress-induced cell death. Mol Biol Cell 2011; 22: 128–140. 21119000 10.1091/mbc.E10-06-0556 PMC3016971

[bib16] Gonzalvez F, Lawrence D, Yang B, Yee S, Pitti R, Marsters S et al. TRAF2 Sets a threshold for extrinsic apoptosis by tagging caspase-8 with a ubiquitin shutoff timer. Molecular Cell 2012; 48: 888–899. 23142077 10.1016/j.molcel.2012.09.031

[bib17] Tzeng HP, Evans S, Gao F, Chambers K, Topkara VK, Sivasubramanian N et al. Dysferlin mediates the cytoprotective effects of TRAF2 following myocardial ischemia reperfusion injury. J Am Heart Assoc 2014; 3: e000662. 24572254 10.1161/JAHA.113.000662 PMC3959693

[bib18] Piao JH, Hasegawa M, Heissig B, Hattori K, Takeda K, Iwakura Y et al. Tumor necrosis factor receptor-associated factor (TRAF) 2 controls homeostasis of the colon to prevent spontaneous development of murine inflammatory bowel disease. J Biol Chem 2011; 286: 17879–17888. 21393251 10.1074/jbc.M111.221853 PMC3093863

[bib19] Shen HM, Lin Y, Choksi S, Tran J, Jin T, Chang L et al. Essential roles of receptor-interacting protein and TRAF2 in oxidative stress-induced cell death. Mol Cell Biol 2004; 24: 5914–5922. 15199146 10.1128/MCB.24.13.5914-5922.2004 PMC480890

[bib20] Thomas GS, Zhang LQ, Blackwell K, Habelhah H. Phosphorylation of TRAF2 within its RING domain inhibits stress-induced cell death by promoting IKK and suppressing JNK activation. Cancer Res 2009; 69: 3665–3672. 19336568 10.1158/0008-5472.CAN-08-4867 PMC2669835

[bib21] Chen Z, Wang G, Zhai X, Hu Y, Gao D, Ma L et al. Selective inhibition of protein kinase C beta2 attenuates the adaptor P66 Shc-mediated intestinal ischemia-reperfusion injury. Cell Death Dis 2014; 5: e1164. 24722289 10.1038/cddis.2014.131 PMC5424109

[bib22] Butler AM, Buzhardt MLS, Erdogan E, Li SH, Inman KS, Fields AP et al. A small molecule inhibitor of atypical protein kinase C signaling inhibits pancreatic cancer cell transformed growth and invasion. Oncotarget 2015; 6: 15297–15310. 25915428 10.18632/oncotarget.3812 PMC4558152

[bib23] Siddiqi S, Mansbach CM 2nd. Dietary and biliary phosphatidylcholine activates PKCzeta in rat intestine. J Lipid Res 2015; 56: 859–870. 25713101 10.1194/jlr.M056051 PMC4373743

[bib24] Chen Y, Lui VC, Rooijen NV, Tam PK. Depletion of intestinal resident macrophages prevents ischaemia reperfusion injury in gut. Gut 2004; 53: 1772–1780. 15542513 10.1136/gut.2003.034868 PMC1774329

[bib25] Wu B, Qiu W, Wang P, Yu H, Cheng T, Zambetti GP et al. p53 independent induction of PUMA mediates intestinal apoptosis in response to ischaemia-reperfusion. Gut 2007; 56: 645–654. 17127703 10.1136/gut.2006.101683 PMC1942137

[bib26] Zhou J, Huang WQ, Li C, Wu GY, Li YS, Wen SH et al. Intestinal ischemia/reperfusion enhances microglial activation and induces cerebral injury and memory dysfunction in rats. Crit Care Med 2012; 40: 2438–2448. 22647410 10.1097/CCM.0b013e3182546855

[bib27] Higuchi S, Wu R, Zhou M, Marini CP, Ravikumar TS, Wang P. Gut hyperpermiability after ischemia and reperfusion: attenuation with adrenomedullin and its binding protein treatment. Int J Clin Exp Pathol 2008; 1: 409–418. 18787625 PMC2480576

[bib28] Tendler DA. Acute intestinal ischemia and infarction. Semin Gastrointest Dis 2003; 14: 66–76. 12889581

[bib29] Farber A, Connors JP, Friedlander RM, Wagner RJ, Powell RJ, Cronenwett JL. A specific inhibitor of apoptosis decreases tissue injury after intestinal ischemia-reperfusion in mice. J Vasc Surg 1999; 30: 752–760. 10514215 10.1016/s0741-5214(99)70115-1

[bib30] Kanzaki M, Mora S, Hwang JB, Saltiel AR, Pessin JE. Atypical protein kinase C (PKCzeta/lambda) is a convergent downstream target of the insulin-stimulated phosphatidylinositol 3-kinase and TC10 signaling pathways. J Cell Biol 2004; 164: 279–290. 14734537 10.1083/jcb.200306152 PMC2172328

[bib31] Standaert ML, Bandyopadhyay G, Kanoh Y, Sajan MP, Farese RV. Insulin and PIP3 activate PKC-zeta by mechanisms that are both dependent and independent of phosphorylation of activation loop (T410) and autophosphorylation (T560) sites. Biochemistry 2001; 40: 249–255. 11141077 10.1021/bi0018234

[bib32] Fuly AL, Machado AL, Castro P, Abrahao A, Redner P, Lopes UG et al. Lysophosphatidylcholine produced by the phospholipase A2 isolated from Lachesis muta snake venom modulates natural killer activity as a protein kinase C effector. Toxicon 2007; 50: 400–410. 17537472 10.1016/j.toxicon.2007.04.008

[bib33] Scott GA, Arioka M, Jacobs SE. Lysophosphatidylcholine mediates melanocyte dendricity through PKCzeta activation. J Invest Dermatol 2007; 127: 668–675. 17024099 10.1038/sj.jid.5700567

[bib34] Regala RP, Thompson EA, Fields AP. Atypical protein kinase C iota expression and aurothiomalate sensitivity in human lung cancer cells. Cancer Res 2008; 68: 5888–5895. 18632643 10.1158/0008-5472.CAN-08-0438 PMC2662432

[bib35] Moscat J, Diaz-Meco MT. The atypical protein kinase Cs. Functional specificity mediated by specific protein adapters. EMBO Rep 2000; 1: 399–403. 11258478 10.1093/embo-reports/kvd098 PMC1083770

[bib36] Qiu RG, Abo A, Steven Martin G. A human homolog of the C. elegans polarity determinant Par-6 links Rac and Cdc42 to PKCzeta signaling and cell transformation. Curr Biol 2000; 10: 697–707. 10873802 10.1016/s0960-9822(00)00535-2

[bib37] Grunicke HH, Spitaler M, Mwanjewe J, Schwaiger W, Jenny M, Ueberall F. Regulation of cell survival by atypical protein kinase C isozymes. Adv Enzyme Regul 2003; 43: 213–228. 12791393 10.1016/s0065-2571(02)00032-8

[bib38] Murray NR, Jamieson L, Yu W, Zhang J, Gokmen-Polar Y, Sier D et al. Protein kinase Ciota is required for Ras transformation and colon carcinogenesis *in vivo*. J Cell Biol 2004; 164: 797–802. 15024028 10.1083/jcb.200311011 PMC2172278

[bib39] Erdogan E, Lamark T, Stallings-Mann M, Lee J, Pellecchia M, Thompson EA et al. Aurothiomalate inhibits transformed growth by targeting the PB1 domain of protein kinase Ciota. J Biol Chem 2006; 281: 28450–28459. 16861740 10.1074/jbc.M606054200

[bib40] Li S, Wang L, Dorf ME. PKC phosphorylation of TRAF2 mediates IKKalpha/beta recruitment and K63-linked polyubiquitination. Mol Cell 2009; 33: 30–42. 19150425 10.1016/j.molcel.2008.11.023 PMC2643372

[bib41] Shen RR, Zhou AY, Kim E, Lim E, Habelhah H, Hahn WC. IkappaB kinase epsilon phosphorylates TRAF2 to promote mammary epithelial cell transformation. Mol Cell Biol 2012; 32: 4756–4768. 23007157 10.1128/MCB.00468-12 PMC3497603

[bib42] Jackson-Bernitsas DG, Ichikawa H, Takada Y, Myers JN, Lin XL, Darnay BG et al. Evidence that TNF-TNFR1-TRADD-TRAF2-RIP-TAK1-IKK pathway mediates constitutive NF-kappaB activation and proliferation in human head and neck squamous cell carcinoma. Oncogene 2007; 26: 1385–1397. 16953224 10.1038/sj.onc.1209945

[bib43] Zhou AY, Shen RR, Kim E, Lock YJ, Xu M, Chen ZJ et al. IKKepsilon-mediated tumorigenesis requires K63-linked polyubiquitination by a cIAP1/cIAP2/TRAF2 E3 ubiquitin ligase complex. Cell Rep 2013; 3: 724–733. 23453969 10.1016/j.celrep.2013.01.031 PMC4135466

[bib44] Petersen SL, Chen TT, Lawrence DA, Marsters SA, Gonzalvez F, Ashkenazi A. TRAF2 is a biologically important necroptosis suppressor. Cell Death Differ 2015; 22: 1846–1857. 25882049 10.1038/cdd.2015.35 PMC4648330

[bib45] Tai DI, Tsai SL, Chen YM, Chuang YL, Peng CY, Sheen IS et al. Activation of nuclear factor kappaB in hepatitis C virus infection: implications for pathogenesis and hepatocarcinogenesis. Hepatology 2000; 31: 656–664. 10706556 10.1002/hep.510310316

[bib46] Lin S, Hoffmann K, Gao C, Petrulionis M, Herr I, Schemmer P. Melatonin promotes sorafenib-induced apoptosis through synergistic activation of JNK/c-jun pathway in human hepatocellular carcinoma. J Pineal Res 2017; 62. 10.1111/jpi.12398 28178378

[bib47] Tracey L, Perez-Rosado A, Artiga MJ, Camacho FI, Rodriguez A, Martinez N et al. Expression of the NF-kappaB targets BCL2 and BIRC5/Survivin characterizes small B-cell and aggressive B-cell lymphomas, respectively. J Pathol 2005; 206: 123–134. 15880597 10.1002/path.1768

[bib48] Turco MC, Romano MF, Petrella A, Bisogni R, Tassone P, Venuta S. NF-kappaB/Rel-mediated regulation of apoptosis in hematologic malignancies and normal hematopoietic progenitors. Leukemia 2004; 18: 11–17. 14574329 10.1038/sj.leu.2403171

[bib49] Imahashi K, Schneider MD, Steenbergen C, Murphy E. Transgenic expression of Bcl-2 modulates energy metabolism, prevents cytosolic acidification during ischemia, and reduces ischemia/reperfusion injury. Circ Res 2004; 95: 734–741. 15345651 10.1161/01.RES.0000143898.67182.4c

[bib50] Tanaka M, Nakae S, Terry RD, Mokhtari GK, Gunawan F, Balsam LB et al. Cardiomyocyte-specific Bcl-2 overexpression attenuates ischemia-reperfusion injury, immune response during acute rejection, and graft coronary artery disease. Blood 2004; 104: 3789–3796. 15280201 10.1182/blood-2004-02-0666

[bib51] Krishnan S, Intlekofer KA, Aggison LK, Petersen SL. Central role of TRAF-interacting protein in a new model of brain sexual differentiation. Proc Natl Acad Sci USA 2009; 106: 16692–16697. 19805359 10.1073/pnas.0906293106 PMC2757835

[bib52] Baud V, Liu ZG, Bennett B, Suzuki N, Xia Y, Karin M. Signaling by proinflammatory cytokines: oligomerization of TRAF2 and TRAF6 is sufficient for JNK and IKK activation and target gene induction via an amino-terminal effector domain. Genes Dev 1999; 13: 1297–1308. 10346818 10.1101/gad.13.10.1297 PMC316725

[bib53] Chiu CJ, McArdle AH, Brown R, Scott HJ, Gurd FN. Intestinal mucosal lesion in low-flow states. I. A morphological, hemodynamic, and metabolic reappraisal. Arch Surg 1970; 101: 478–483. 5457245 10.1001/archsurg.1970.01340280030009

