# Supplementary material for: PKCζ phosphorylates TRAF2 to protect against intestinal ischemia–reperfusion–induced injury
Source: Cell Death Dis. 2017 Jul 20;8(7):e2935–. doi: 10.1038/cddis.2017.310 (PMC5550857; doi:10.1038/cddis.2017.310)
Supplement: Supplementary Figure S1 [file cddis2017310x1.doc]

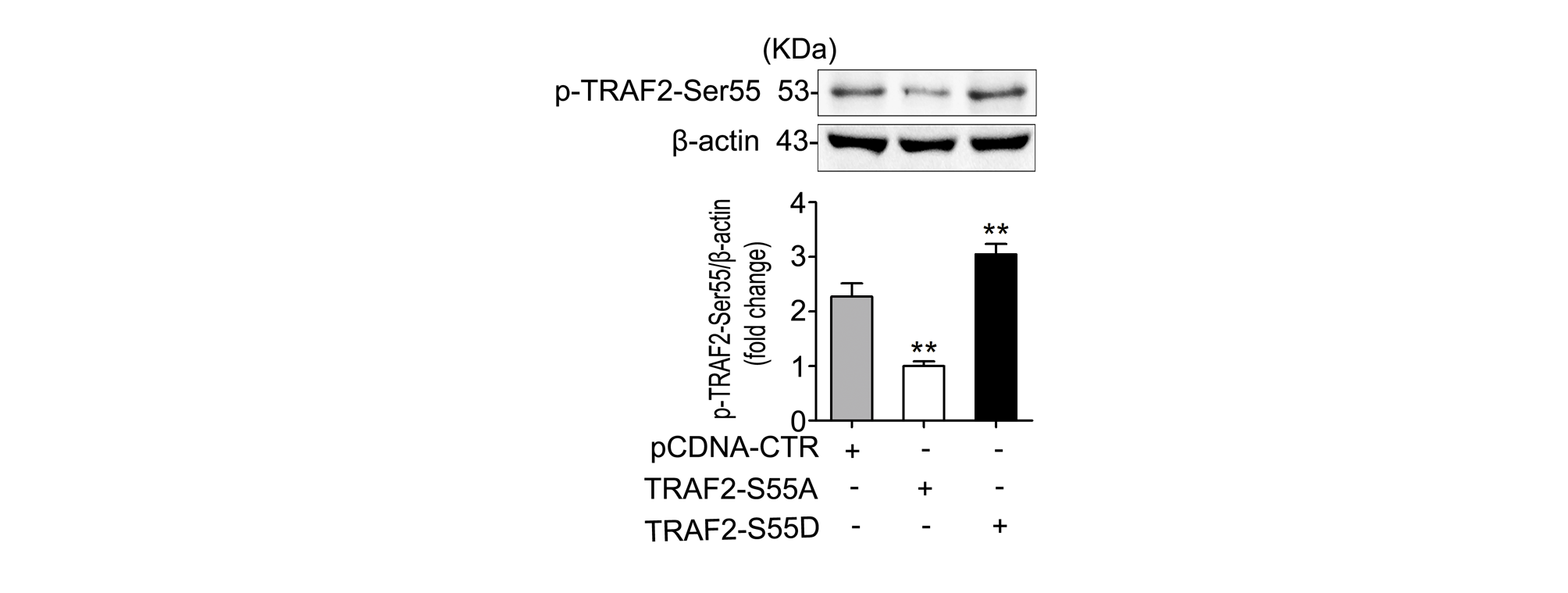


**Supplementary Figure S1 Phospho-mutant TRAF2 plasmids affect the expression of p-TRAF2-Ser55 protein.**

Caco-2 cells were transfected with an empty vector plasmid (pCDNA-CTR), TRAF2-S55A or TRAF2-S55D. Western blotting demonstrated the expression of p-TRAF2-Ser55. β-actin was included as a loading control. The data are shown as the means ± S.D. (n = 3). **P < 0.01 versus pCDNA-CTR.
